# Disseminated Herpes Simplex Virus with Fulminant Hepatitis

**DOI:** 10.1155/2015/463825

**Published:** 2015-07-28

**Authors:** Bassam H. Rimawi, Joseph Meserve, Ramzy H. Rimawi, Zaw Min, John W. Gnann

**Affiliations:** ^1^Division of Maternal Fetal Medicine and Reproductive Infectious Diseases, Department of Gynecology and Obstetrics, Emory University School of Medicine, 550 Peachtree Street, 8th Floor, Atlanta, GA 30303, USA; ^2^Division of Infectious Diseases, Department of Internal Medicine, Medical University of South Carolina, Charleston, SC 29403, USA; ^3^Division of Critical Care and Infectious Diseases, Department of Internal Medicine, Emory University School of Medicine, 550 Peachtree Street, Atlanta, GA 30303, USA; ^4^Division of Infectious Diseases, Department of Medicine, Temple University School of Medicine, 420 East North Avenue, East Wing, Suite 407, Pittsburgh, PA 15212, USA

## Abstract

Disseminated herpes simplex virus (HSV) is a rare cause of acute fulminant liver failure. We hereby present a case series of three patients with acute disseminated HSV with necrotizing hepatitis successfully treated with a week course of acyclovir. Early empiric administration of acyclovir therapy while awaiting confirmatory tests is critical in this potentially lethal disease.

## 1. Case 1

**Figure 1:**
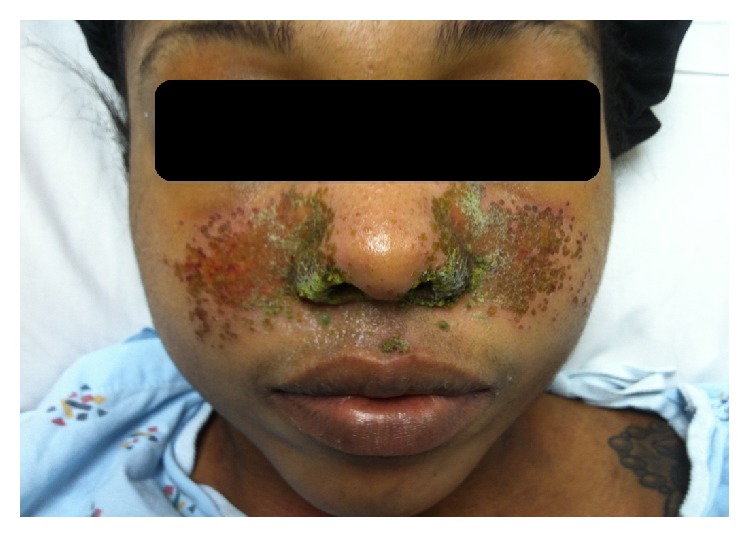
Case 1 demonstrates facial eczema herpeticum.

A 25-year-old nonpregnant woman presented with a two-week history of fever, chills, myalgias, and fatigue. She denied any relevant medical history, including sexually transmitted infections. She reported 6 lifetime male sexual partners with inconsistent condom use. On physical examination, her temperature was 100.2°F and she had an erythematous rash with papules and scaling over the malar region of her face, consistent with eczema herpeticum, Figure 1. She did not have any predisposing skin conditions for the development of eczema herpeticum, for example, atopic dermatitis. With respect to her serum-antibody status, her HSV-2 immunoglobulin M (IgM) was positive with a negative immunoglobulin G (IgG). Her serologies for HSV-1 (IgM and IgG) were both negative. Her genital examination consisted of multiple, tender, small ulcerations over her labia and perineum. Her liver function tests were elevated, Table 1. Human immunodeficiency virus (HIV) serology was negative. Suspicion for acute necrotizing HSV hepatitis led to the empiric initiation of acyclovir 10 mg/kg intravenous every 8 hours. Viral culture of her genital lesions was positive for HSV-2. Quantitative HSV-2 polymerase chain reaction (PCR) of her blood demonstrated 1.1 million copies/mL, which declined to less than 100,000 copies/mL after 5 days of acyclovir therapy. She completed a 7-day course of intravenous acyclovir with good response and a negative follow-up HSV cerebrospinal fluid (CSF) PCR. Her laboratory findings and physical examination were normal at her 1-month follow-up visit. Seroconversion of her serum-antibody status demonstrated that she acquired a primary infection in that her HSV-2 IgM became negative after treatment and her HSV-2 IgG was positive.

## 2. Case 2

**Figure 2:**
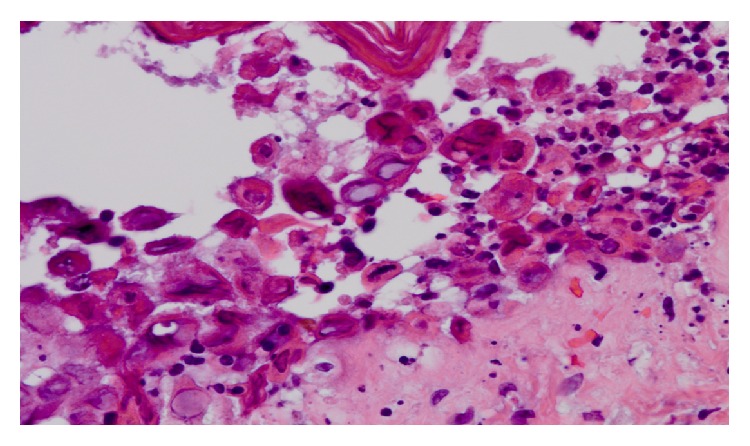
Case 2 demonstrating intraepidermal vesiculation with associated acantholysis and multinucleated cells characteristic of herpes simplex virus.

A 24-year-old nonpregnant woman presented with a 10-day history of intermittent fevers, chills, nausea, and abdominal pain. She denied any past medical history, including sexually transmitted infections. She reported 4 lifetime male sexual partners and was in a stable monogamous relationship without the use of contraception at the time of her presentation. Pertinent findings included a fever of 101.1°F, bilateral scleral icterus, and 12 small papulovesicular lesions on her trunk and extremities. Skin biopsies demonstrated multinucleated cells characteristic of HSV, Figure 2. A presumptive diagnosis of acute necrotizing HSV hepatitis was made and intravenous acyclovir was empirically initiated. With respect to her serum-antibody status, her HSV-2 IgM was positive with a negative HSV-2 IgG. Her serologies for HSV-1 (IgM and IgG) were both negative. Quantitative HSV-2 PCR of blood demonstrated 500 million copies/mL, which declined to 400,000 copies/mL after 6 days of antiviral therapy. She completed a 7-day course of intravenous acyclovir with good response and a negative follow-up HSV CSF PCR. Her liver function tests returned to normal, and her cutaneous skin lesions had resolved by her 1-month follow-up visit. Seroconversion of her serum-antibody status demonstrated that she acquired a primary infection in that her HSV-2 IgM became negative after treatment and her HSV-2 IgG was positive.

## 3. Case 3

**Figure 3:**
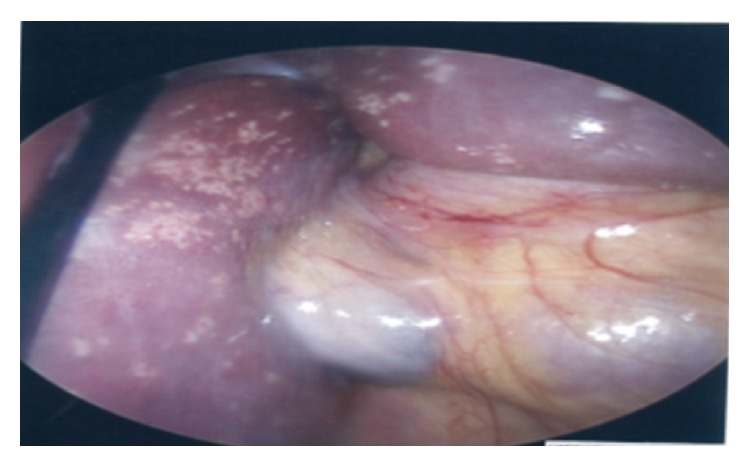
Case 3 demonstrates multiple white raised irregularly shaped lesions on the surface of the liver that were noted at the time of a diagnostic laparoscopy, with biopsies consistent with HSV-2 hepatitis and confirmed with immunofluorescent staining of the tissue culture cells.

A 27-year-old pregnant woman, gravida 1 para 0, at 23 weeks and 2 days of gestation, presented with a five-day history of epigastric and right upper quadrant (RUQ) abdominal pain. She denied any significant past medical history, including sexually transmitted infections. She had marked RUQ tenderness and a single, solitary, nontender, nonulcerative, erythematous, vesicular lesion within the vaginal introitus just below the clitoris. She lacked lymphadenopathy or other pertinent physical examination findings. With respect to her serum-antibody status, her HSV-2 IgM was positive with a negative HSV-2 IgG. Her serologies for HSV-1 (IgM and IgG) were both negative. A viral culture and histopathologic examination using immunofluorescent staining of vaginal vesicular lesion were consistent with HSV-2. Diagnostic laparoscopy demonstrated multiple, white, raised, irregularly shaped lesions on the surface of her liver. Biopsies, confirmed with immunofluorescent staining of the tissue culture cells, were consistent with HSV-2 hepatitis, Figure 3. Intravenous acyclovir 10 mg/kg every 8 hours was initiated, though her hospital course was complicated by HSV encephalitis. Magnetic resonance imaging of her brain showed an enhancing signal in the right temporal lobe. PCR testing of her CSF was positive for HSV-2. She subsequently went into preterm labor and delivered a 23-week fetus with multiple desquamating and vesicular lesions on both lower extremities and midback, of which viral culture confirmed HSV-2. The fetus unfortunately demised within 2 hours after birth. Her mental status improved by day 7 of intravenous acyclovir. She completed a 21-day course with normalization of her liver function tests, negative follow-up HSV CSF PCR, and resolution of her cutaneous skin lesions at her 1-month follow-up visit. Seroconversion of her serum-antibody status demonstrated that she acquired a primary infection in that her HSV-2 IgM became negative after treatment and her HSV-2 IgG was positive.

## 4. Discussion

Since it was first described in 1969, HSV remains a rare, yet potentially lethal, cause of acute necrotizing hepatitis [1]. HSV reportedly causes 0.8% of acute liver failure and 2–4% of acute viral hepatitis [2]. Although both HSV-1 and HSV-2 have been reported to cause similar complications, our case series describes three patients with sexually acquired genital HSV-2, complicated by necrotizing herpes hepatitis. Despite its complications, acyclovir can offer promising results as we have described.

HSV hepatitis is a condition that can lead to fulminant hepatic necrosis with 100- to 1,000-fold rises in serum aminotransferase levels [2]. Though immunocompetent patients are at risk, mortality rates of up to 75% have been reported, with the majority of cases described in immunocompromised hosts (organ transplant and HIV patients) and pregnant women (primarily in the third trimester) [3, 4]. A decrease in the immunoglobulin G, from 27 through 33 weeks of gestation, correlates with the 20–30% hemodilution that occurs in pregnancy at that time [2].

Although all our patients had mucocutaneous lesions consistent with HSV, this is not a universal finding and the absence of suggestive mucocutaneous lesions does not exclude HSV hepatitis [3]. While liver biopsy has historically been the gold standard for diagnosis of HSV hepatitis, serum HSV PCR is becoming the diagnostic modality of choice with a high sensitivity and specificity [4]. Also of importance, all three patients demonstrated a positive HSV-2 IgM and a negative HSV-2 IgG at the time of their presentation, with seroconversion of their serum-antibody status, consistent with a primary infection, hence the viremia that was noted in these cases.

A delay in the diagnosis and initiation of antiviral therapy contributes to a poor outcome with rapid sequelae [5]. Unfortunately, due to the nonspecific clinical presentation, absence of mucocutaneous findings, and lack of a rapid diagnostic assay, the diagnosis is often made postmortem [3]. The diagnosis was reportedly considered antemortem in just 33% of pregnant patients and 26% of nonpregnant patients [6].

Early treatment with acyclovir can inhibit viral replication and enhance the probability of survival [7]. Healthcare providers should be aware of HSV hepatitis in patients with elevated liver enzymes even in the absence of suggestive cutaneous features, as empiric therapy can prevent further morbidity and mortality. Given the frequent delay in diagnosis, low risk-benefit ratio, and significantly improved outcomes, we recommend empiric acyclovir therapy for patients presenting with acute liver failure of unknown etiology until HSV hepatitis can be excluded, especially in immunocompromised hosts and pregnant women.

A 5–7-day course of intravenous acyclovir is currently recommended for most herpes simplex infections, unless HSV encephalitis is diagnosed in which treatment should be extended to 21 days [8]. With respect to our three presenting patients, the first two cases were diagnosed with HSV hepatitis and clearly showed improvement within 5-6 days of intravenous acyclovir therapy that was initiated promptly after diagnosis. These two cases completed a 7-day course and both had complete resolution of their cutaneous lesions and complete normalization of their liver function tests at their one-month follow-up visit.

The third case had a more aggressive HSV involvement, having both HSV hepatitis and HSV encephalitis, with notable dissemination across intact amniotic membranes to her fetus. The fetus demonstrated typical multiple desquamating and vesicular lesions on both lower extremities and midback consistent with HSV-2 involvement and demised within 2 hours after birth, likely from a combination of very early preterm birth and infectious sequelae. Despite this disease severity, our third subject still demonstrated rapid improvement by day 7 of therapy. By 3 weeks of therapy, she had complete resolution of her cutaneous vaginal lesion and liver function tests.

In conclusion, HSV hepatitis remains a rare, yet deadly, disease if not diagnosed and treated early. We hereby present three cases of HSV hepatitis successfully treated due to a high, early index of suspicion and treatment with intravenous acyclovir therapy. Given its rapid complication rates, we recommend initiating antiviral therapy empirically while awaiting HSV testing.

## Figures and Tables

**Table 1:** Patient's laboratory results.

Lab values	AST^a^ (10–30 U/L)^d^	ALT^b^ (7–35 U/L)^d^	Alkaline phosphatase (25–100 U/L)^d^	Total bilirubin (0.3–1.2 mg/dL)^d^	Platelets (150–450 ×10^3^/*µ*L)^d^	INR^c^ (0.8–1.2)^d^	HSV^e^	
							PCR^f^	
							Maternal serum (copies/mL)	
							Before therapy	After therapy
Case 1	3685	4101	114	2.1	278	1.69	1.1 million	100,000
Case 2	2075	3886	203	11	140	3.6	500 million	400,000
Case 3	1902	731	127	2.0	90	3.2	NP^*∗*^	NP

U/L: units per liter.

^a^Aspartate aminotransferase.

^b^Alanine aminotransferase.

^c^International normalized ratio.

^d^Normal reference range.

^e^Herpes simplex virus.

^f^Polymerase chain reaction.

^*∗*^NP: not performed.
